# Developing management pathways for hypertensive disorders of pregnancy (HDP) in Indonesian primary care: a study protocol

**DOI:** 10.1186/s12978-019-0674-0

**Published:** 2019-02-01

**Authors:** Fitriana Murriya Ekawati, Sharon Licqurish, Ova Emilia, Jane Gunn, Shaun Brennecke, Phyllis Lau

**Affiliations:** 1 Department of Family and Community Medicine, Universitas Gadjah Mada, Yogyakarta, Indonesia; 2 Department of General Practice, University of Melbourne, 200 Berkeley St, Carlton, Vic 3053 Australia; 3 School of Nursing and Midwifery, Monash University, Clayton, Vic Australia; 4 Department of Obstetrics and Gynaecology, Universitas Gadjah Mada/Sardjito Hospital, Yogyakarta, Indonesia; 5 University of Melbourne Department of Obstetrics and Gynaecology, Royal Women’s Hospital, Parkville, Victoria Australia; 6 Pregnancy Research Centre, Department of Maternal-Fetal Medicine, Royal Women’s Hospital, Parkville, Victoria Australia

**Keywords:** Hypertension, Pregnancy, Pilot study, Implementation science, Delphi technique, Primary care, Feasibility, Acceptability

## Abstract

**Background:**

National and international guidelines for the management of hypertensive disorders of pregnancy (HDP) are available in developing countries. However, more detailed clinical pathways for primary care settings are limited. This study focuses on Indonesia, where 72% of women who died from HDP and its complications had received less appropriate treatment according to international guidelines. There is an urgent need to develop primary care focused pathways that enable general practitioners (GPs), midwives and other relevant providers to manage HDP better.

**Objectives:**

This paper describes a study protocol for the development of HDP management pathways for Indonesian primary care settings.

**Methods:**

This study design is informed by Implementation Science theories and consists of three phases. The exploratory phase will involve conducting semi-structured interviews with key Indonesian primary care stakeholders to explore their experiences and views on HDP management. The development phase will apply evidence from the literature review and results of the exploratory phase to develop HDP management pathways contextualised to Indonesian primary care settings. Consensus will be sought from approximately 50 experts, consist of general practitioners (GPs), midwives, obstetricians, nurses and policy makers using Delphi technique survey. The evaluation phase will involve a pilot study to evaluate the pathways’ acceptability and feasibility in a sample of Indonesian primary care practices using mixed methods.

**Discussion:**

The implementation science frameworks inform and guide the phases in this study. Qualitative interviews in the exploratory phase are conducive to eliciting opinions from key stakeholders. Using Delphi technique at the development phase is suitable to seek participants’ consensus on HDP management in primary care. Observations, focus group discussions, interviews as well as analysis of patients’ medical records at the evaluation phase are expected to provide a comprehensive investigation of the implementation of the pathways in practice settings.

## Plain English summary

This paper describes the study design to develop pathways and recommendations for doctors, nurses and midwives to manage high blood pressure diseases during pregnancy in Indonesia. The study will apply three phases where information from experts will be obtained to inform the pathways development, and then we will trial the pathways in Indonesian primary care settings. Our proposed protocol is suitable more broadly for the development of other clinical pathways in primary care in developing countries.

## Background

Hypertensive disorders of pregnancy (HDP) occur in up to 10% of all pregnancies worldwide and are the second highest cause of maternal mortality [1]. These disorders are characterised by high blood pressure measurement (systolic blood pressure > 140 mmHg and diastolic blood pressure > 90 mmHg) in pregnant women, and are classified into the diagnosis of chronic hypertension, gestational hypertension, masked hypertension, white coat hypertension and preeclampsia [2]. The complication of HDP can be life-threatening for the affected women, including the risks of stroke, liver and kidney failure. The World Health Organization (WHO) reported that HDP and its complication has caused up to 14% of the global maternal deaths, or more than 70,000 cases per year [2, 3] and almost of them occur in developing countries [3]. In Indonesia, more than 1000 maternal deaths or a third of the country’s total maternal mortality were attributed to HDP [4] and made it the highest among South East Asia [5].

Primary care plays essential roles to provide maternal care in many developing countries. It is normally the settings where patients see the health care provider for the first time in the health system [6, 7] and is the most convenient health facilities near to their residence [8]. In Indonesia, primary care is accessed by more than 90% of pregnant women throughout their pregnancy; it provides help with the delivery and follow-up care after delivery in hospitals [5, 9]. However, HDP cases were often detected late in the primary care setting and subsequently affect timely referral to secondary care. Initial treatment before referral was also unstandardized often causing the affected women’s condition to be too critical by the time they reach hospitals [4, 10–12].

To improve HDP management in Indonesian primary care, primary care providers’ practice related to HDP needs to be upgraded. A potential way is to establish viable clinical pathways. Whilst national and international HDP guidelines are available in Indonesia [13–16], the guidelines’ contents are general and predominantly designed for specialist and hospital settings. Their implementation in primary care is often challenged by different practice environtment, limited facilities and providers’ knowledge. Therefore, the guidelines need to be contextualised and be more appropriate to the Indonesian primary care settings so that they are useable and able to assist primary care providers to better manage HDP cases [17, 18].

This paper describes the research design and protocol to develop and pilot test primary care HDP management pathways for Indonesia. The study aims to contextualise evidence-based HDP management guidelines to the Indonesian primary care settings to facilitate prevention, detection and appropriate referral for women in order to reduce avoidable maternal mortality associated with HDP.

## Methods/design

### Theoretical frameworks

**Fig. 1:**
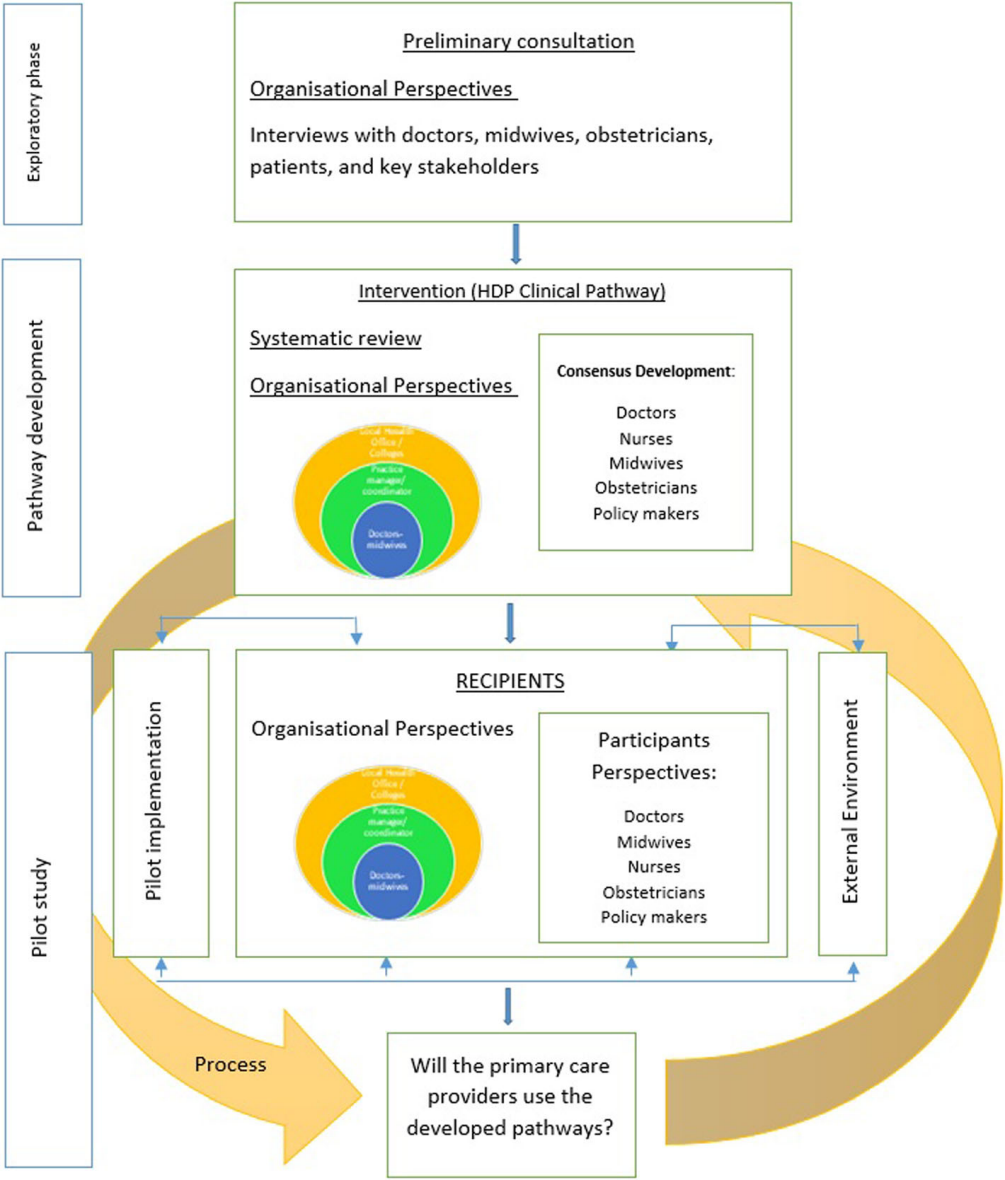
Theoretical framework adapted from an amalgamation of the MRC and PRISM frameworks

**Fig. 2:**
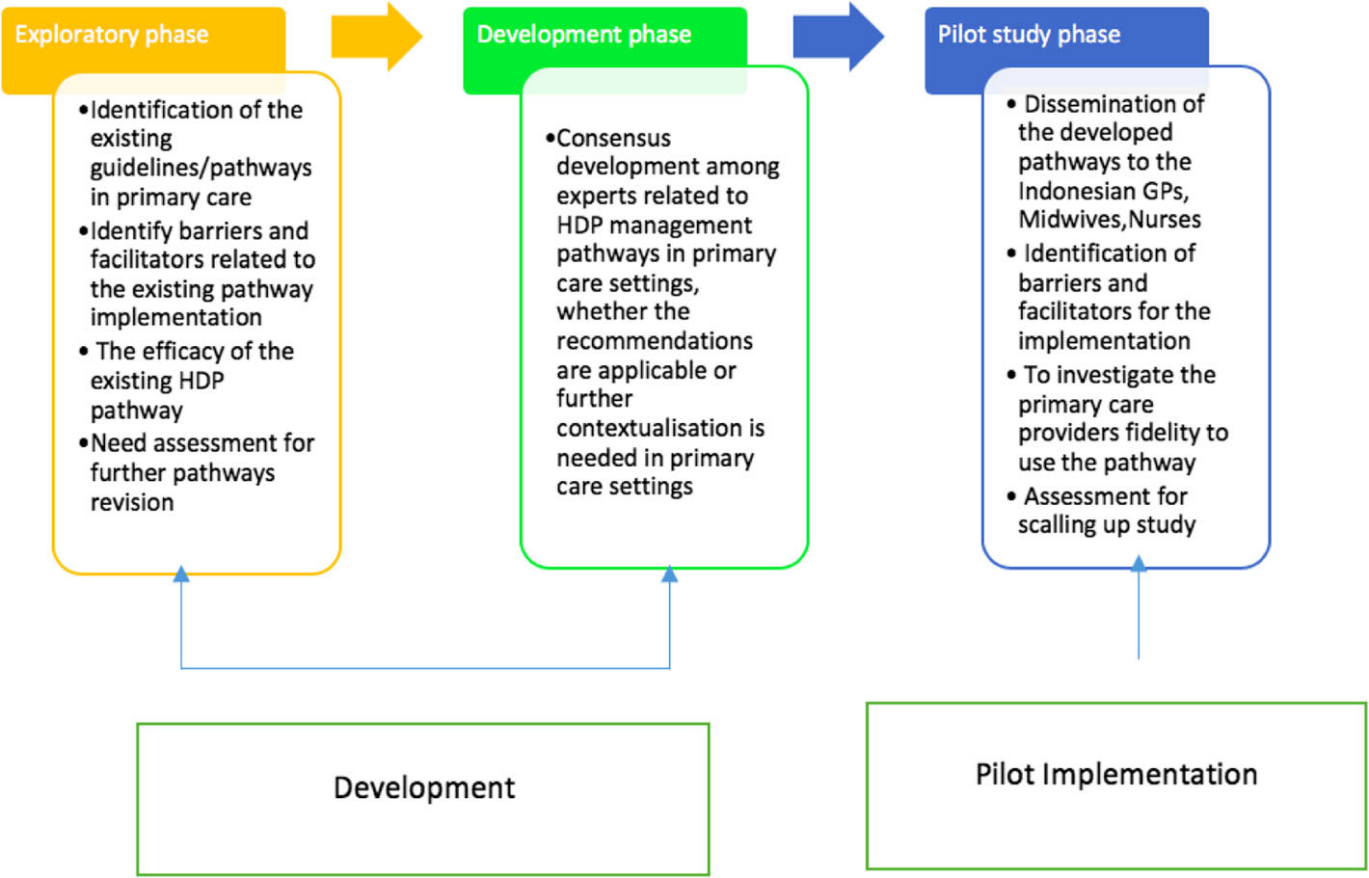
Design of the study phases

This study is informed by an amalgamation of the the United Kingdom (UK) Medical Research Council (MRC) framework for developing and evaluating complex interventions [19] and the Practical, Robust, Implementation and Sustainability Model (PRISM) [20] (Fig. 1). The MRC framework gave practical guidance on essential steps for planning, developing and evaluating an intervention implementation, e.g. the critical factors that needed to be explored before developing an intervention and the need to pilot an intervention before implementing it in practice [19]. PRISM was proposed by Feldstein and Glasgow [20] as a model to develop and evaluate an intervention, with a focus on the organizational characteristics of the implementation setting as well as the extrinsic factors associated with the implementation. The resultant theoretical framework from the combination of MRC and PRISM frameworks is shown in Fig. 2.

#### Study setting

Indonesia is the fourth most populous country in the world with more than 265 million people in 2015 [21]. Maternal mortality in this country is very high – approximately 359 deaths per 100,000 live births in 2015 [22] and HDP is responsible for up to a third of the cases [23].

This study will focus in Yogyakarta province in Indonesia. This province is located in Java island and its population was 3,720,912 in 2017. This province was one of the most populous province in the country [24]. Similar to other provinces, Yogyakarta has a stratified health system level consisting of primary-secondary-and tertiary health care. Primary care is provided by public primary care clinics (Puskesmas), midwives’ practices or private clinics, while secondary and tertiary care are provided at the secondary and tertiary hospitals. Maternal health care is nowadays funded under a public insurance scheme (*Jaminan Kesehatan Nasional*/JKN) [25], with an additional co-funding scheme from local government insurance *(Jaminan Kesehatan Daerah/Jamkesda)* [8, 26, 27]*.* Compared to the other provinces, Yogyakarta has a relatively good health care access and superiorly has an available local referral manual for the management of pregnancy complications [28]. However still, maternal mortality from HDP related complications remains high [29], and more detailed pathways for HDP management in primary care is limited [28].

### Exploratory phase

This phase aims to explore the views and experience of Indonesian GPs, nurses, midwives, obstetricians and policymakers related to current management of HDP, HDP guideline being used in practice, and the barriers and facilitators to optimal HDP management in primary care settings. The results of this phase will inform the development and pilot phases.

### Design and participants’ recruitment

Approximately 30 participants consisting of general practitioners (GPs) (*n* = 5), nurses (*n* = 5), midwives (*n* = 5), obstetricians (*n* = 5), women with a history of HDP (*n* = 5) and policymakers (*n* = 5) will be invited to participate. All prospective participants, except women with HDP history are identified through the researchers’ professional networks and via snowballing recruitment. All prospective health professional and policymaker participants will be emailed or phone contacted regarding this study, while the women with a history of HDP will be recruited through the GPs or midwives involved. All participants will be provided with a-Bahasa Indonesia plain language statement (PLS) and asked to provide written/oral consent prior to the interview. The development of the interview guides is informed by domains of the project’s theoretical frameworks, and the interviews are conducted in Bahasa Indonesia. The English version of the interview guides is presented in Table 1.

**Table 1:** Guiding questions for interviews in exploratory phase

Project theoretical frameworks	Group of participants involved in the exploratory phase	Guiding questions for the participants
Intervention	Primary care providers	How is HDP usually managed in primary care?
Recipients’ (individuals involved in intervention implementation) characteristic	Obstetricians	What do you think about HDP management in primary care?
	Policy makers & key informants	
		What are the current guidelines to manage hypertension in pregnancy in primary care?
	Women	
		What are things work well?
		What are things need to be improved?
		What is your experience of having HDP treatment in primary care?
External Environment		
		What are things work well?
		What are things need to be improved?
*Implementation*		

### Data analysis

The interview data will be analysed thematically using a mix of an inductive and deductive approach [30]:

1. All the interviews will be audio-recorded and transcribed. A quarter of the transcripts will be translated into English and back-translated to Bahasa Indonesia for language validation purposes.
2. The first author will read all the transcripts until familiar with the participants’ answers.
3. All significant quotes will be coded with the aid of the NVIVO software [31].
4. Other authors in this study will separately code a subset of the interviews and will discuss the quotes collectively to resolve differences until a consensus is reached.
5. Patterns are then identified from the codes and grouped according to the domains of the project theoretical framework (deductive approach) as well as to elicit emerging new themes (inductive approach).

## Development phase

### Reviews of national and international HDP guidelines

Between August 2017 and June 2018, reviews of academic and grey literature were conducted to identify existing HDP guidelines and evidence-based recommendations for HDP management both published in English and in Bahasa Indonesia between 2007 and 2018 (Table 2). The guidelines were compared and scopes for improvement for HDP management in Indonesia were identified (Table 3). These will be used to inform the development and analysis of the consensus statements in the development phase.

**Table 2:** Lists of reviewed international and Indonesian HDP guidelines

International Guidelines	Country of origin	Indonesian Guidelines
National Institute for Health Care Excellence (NICE) guidelines [42]	United Kingdom	WHO-SEARO Indonesian guidelines for pregnancy complications [16];
Preeclampsia Community Guideline (PRECOG) I [36]	United Kingdom	
PRECOG II [43]	United Kingdom	Yogyakarta referral manuals for pregnancy complication [28];
The European Society of Cardiologists (ESC) [44]	European consensus	Indonesian Obstetrics and Gynaecology Association (POGI) guideline for preeclampsia [53]
Netherland’s multidisciplinary guideline for cardiovascular diseases in pregnancy [45]	Netherland	
American Congress of Obstetricians and Gynecologists (ACOG) [46]	United States	
The American Society of Hypertension (ASH) [47]	United States	
The Society of Obstetric Medicine of Australia and New Zealand (SOMANZ) guideline [48]	Australia	
Queensland State Guideline for HDP [49]	Australia	
The Society of Obstetricians and Gynaecologists of Canada (SOGC) [50]	Canada	
The Association of Ontario Midwives (AOM) guideline on hypertensive disorders of pregnancy [35]	Canada	
The ISSHP recommendation (2018) [2]	International consensus	
France’s consensus statement for hypertensive disorder in pregnancy [51]	France	
WHO recommendation on preeclampsia and eclampsia [52]	International consensus	
WHO section for Hypertensive Disorders of Pregnancy from the WHO guideline on Pregnancy, childbirth, postpartum and newborn care [14]	International consensus	
Hypertension section in the WHO guideline for managing complication in pregnancy [13]	International consensus

**Table 3:** Identified scopes for improvement for Indonesian HDP management following comparison with international guidelines

All pregnant women should have an initial assessment of risk factors of HDP and or preeclampsia, such as previous preeclampsia, chronic hypertension, any related comorbidities, and age during pregnancy.	
The women’s blood pressure should be checked at each antenatal visit.	
Low dose aspirin is prescribed for women with high risks of preeclampsia.	
Low dose aspirin is prescribed for women with high risks of preeclampsia from 12th week of pregnancy until the baby’s delivery.	
Calcium supplementation is prescribed for women with low calcium intake to prevent the event of preeclampsia.	
Women are asked about signs and symptoms of preeclampsia, such as headache, blurred visions, cramps, seizure, eclampsia feet, in each antenatal visit.	
Proteinuria/Dipstick test should be done at least once in each trimester.	
Once the Dipstick test are positive, the pregnant women are checked with any indicators of preeclampsia, such as: kidney function test, complete blood count, and liver function test.	
Once diagnose with preeclampsia, women are assigned for referral.	
Telephone communication with obstetricians prior to the referral to the hospital.	
Paramedic companion during the referral, completed with adequate emergency kits ambulance.	
Antihypertensive agents for the treatment of severe hypertensive disorder of pregnancy, such as calcium channel blockers, or methyldopa-should be available in the practice settings.	
Antihypertensive agents were considered once the women’s blood pressure reached SBP > 150 and or DBP > 100.	
Antihypertensive agents were prescribed without any delays once the women’s blood pressure reached SBP > 160 and or DBP > 110.	
Obstetricians led delivery is booked once women are diagnosed with the hypertensive disorders in pregnancy.	
Monitoring of sign and symptoms of preeclampsia, includes laboratories examination should be available in practice settings.	
Magnesium sulphate IV/IM should be available for the emergency treatment of eclampsia.	
The full regimens of magnesium sulphate IV/IM as treatment for eclampsia seizures.	
Women should be checked for any signs and symptoms of hypertensive disorder of pregnancy (HDP) and or preeclampsia at maximum of six week postpartum in primary care clinics.	
Almost of antihypertensive agents are safe during breastfeeding periods.	
Postpartum counselling about the risks of cardiovascular diseases for women with previous history of HDP.	
Women with history of HDP are offered with non-hormonal contraception.	
Periodic blood measure monitoring and cardiovascular assessment for women with history of HDP	
Counseling about lifestyle modification is offered for women with history of HDP	
Consideration of low dose of aspirin prescription for the next pregnancy.	

Scopes for improvement for the Indonesian HDP pathways have been identified from the review of HDP guidelines (Table 3). However, it is unclear if all of the recommendations are applicable into Indonesian primary care settings. The objective of the development phase is to establish consensus amongst expert participants on the recommendation statements on HDP management that are applicable and whether they need to be contextualised to Indonesian primary care settings. At the end of this phase, a set of HDP management pathways relevant to Indonesian primary care will be developed and ready for the pilot phase.

### Design and participants’ recruitment

Delphi technique was the method chosen for this phase because of its flexibility and ability to offer anonymity to participants. Participants’ information will only be known by the researchers in this study, the participants will also be able to provide their inputs at their convenience and bias of dominant participants can be minimised [32].

Our consensus development will be conducted in up to four online survey rounds with the help of REDcap (Research Electronic Data Capture) platform- a web-based platform to capture research data available for use in University of Melbourne research (https://clinicalresearch.mdhs.unimelb.edu.au/about/health-informatics/redcap).

The first round aims to ask participants about primary care providers’ roles in HDP management and to ask their views and opinions about HDP definition, risks, screening, prevention, and long-term follow up in primary care. Three open questions will also be asked in this round: (i) what are the importance of GPs and other primary care providers roles in HDP management?, (ii) what are what are the potential roles that primary care providers can play in HDP management in primary care? And (iii) what are enabling factors and challenges to manage HDP in Indonesian primary care?

Set of statements informed by the identified scopes for improvement from the review of international and national HDP management guidelines (Table 3) related to HDP definition, risks, screening, prevention, and long-term follow up in primary care will be presented. Participants will be asked to rank the statements in a 5-Likert scales (1 = strongly disagree, 2 = disagree, 3 = neutral, 4 = agree, and 5 = strongly agree), regarding: (i) whether the recommendation is useful to improve care for pregnant women with high blood pressure and or preeclampsia in primary care? and (ii) Whether the recommendation is applicable in the practice settings or need to be further contextualised?

At the end of the survey, participants will be asked whether they have more suggestions for HDP definition, risks, screening, prevention, and long-term follow-up. The summary results of the first round will be presented at the start of the second round, and the participants will be offered the opportunity to change their previous responses if they wish to do so.

The second-round survey aims to achieve consensus opinion on HDP monitoring and management. The summary results from the first-round analysis and a set of recommendations about HDP monitoring and management will be presented to the participants.

The third round aims to finalise the consensus development and seek the participants’ approval for the developed pathway. The final (fourth) round will only be conducted if more than five statements still require consensus testing after round three.

This development phase will aim to recruit approximately 50 participants within the field of HDP management. Inclusion criteria for participants in this phase are:

(i) medical practitioners, researchers and policymakers who work closely in maternal health, such as general practitioners (GPs), midwives, nurses, and obstetricians, local health officers, etc.
(ii) a minimum of two years of working or research experience in maternal health topics;
(iii) a degree in health sciences;
(iv) familiar with the context of primary care in Indonesia or other developing countries settings;
(v) willing to participate in up to four rounds of surveys.

The prospective participants are identified through the authors’ professional networks and snowballing recommendations from participants. International experts (from Australia, USA, etc.) who are familiar with the context of Indonesia or other developing countries will also be included. All potential participants will be emailed and provided with the surveys’ information, aims and procedures as well as the plain language statements and consent pages.

The surveys aims to achieve a minimum of 60% response rate by the end of each survey round [33]. Each statement will need a minimum of 70% agreement from the participants to be regarded as ‘approved for the management pathways’ [32]. The unapproved statements will then be used in the next rounds to allow participants an opportunity to revise their previous responses if they wish to do so until a final consensus is achieved or until the fourth round survey ends.

### Analysis

The qualitative data from the participants will be analised thematically similarly to the methods used for analysis in the exploratory phase. The participant’s demographics and their agreement on the statements will be analysed descriptively using Microsoft Excel software. The aggregate results of the participant’s responses will be analysed for mean, median, standard deviation and interquartile ranges.

## Pilot phase

This phase aims to test the acceptability and feasibility of the developed HDP pathway in primary care settings as well as to gain suggestions to improve the pathways before further scaling up study.

### Design and participants’ recruitment

The design of the pilot phase will depend on the results of the exploratory and development phases; however, the study envisages the steps of the pilot phase to be as below:

- Preparation: this stage includes a discussion between the researchers in this study about the exploratory and development phases’ results and finalise the details of the pilot phase. Ethics application, research registration in Indonesian local health offices as well as the clinics’ recruitment will also be conducted in this stage.
- Capacity building: this stage includes workshops and meetings with the primary care providers in the recruited clinics to inform them about the developed pathways and the pilot study process.
- Pilot implementation: the developed HDP pathways will be implemented for one month in three public primary care clinics in an Indonesian province. The clinics will be required to use the pathways for a minimum of 10 patients (pregnant women or women with a history of HDP) or up to a month period.
- Evaluation: the pilot evaluation will be guided by the project theoretical frameworks. The pilot implementation of the developed pathways will be evaluated using focus group discussions (FGDs), interviews and observations in the primary care clinics. This phase aims to conduct up to ten FGDs consisting of a minimum of two FGDs each with GPs, midwives, nurses and patients; and interviews with local health officers. Schedules to guide focus group discussions and interviews are also informed by the project theoretical frameworks and are listed in Table 4. Observational data will be collected using participant observation methods that allow the researcher to interact with the observed providers in order to seek more understanding about their behaviour and culture [34]. The observation will focus on the primary care providers’ practices in providing maternal care as well as the barriers and facilitators for the pathway’s implementation in the clinics. Observational data will be noted in the field notes.

**Table 4:** Guiding questions for FGD and interviews in pilot phase

Project theoretical framework	Group of participants involved in the pilot phase	Guiding questions for the participants
Intervention	Primary care providers	What do you think about the pathways content?
Recipients’ (individuals involved in intervention implementation) characteristic	Obstetricians	What do you think about the pathways’ application in your clinics?
	Policy makers & key informants	
		What are the supports needed for the pathway implementation?
	Patients	
		What are the barriers and facilitators of the pathways’ implementation?
External Environment		
		What do you think if the pathways need a larger scale trial?
		What do you think about the pathways’ implementation?
*Implementation*		What do you think if the management is applied to other women?
		What are the barriers and facilitators of the management?
		What do you think if the pathways need a larger scale trial?

Clinic and patient level quantitative data will also be gathered from clinic records as a complement to the qualitative evaluation, including:

i. Patient characteristics, such as demographic data and their pregnancy-related morbidities.
ii. Number of patients being diagnosed and managed for HDP, number of referrals, laboratory tests, patient outcomes, and the maternal morbidity and mortality statistics (if applicable).

Up to three clinics in Yogyakarta will be recruited. The prospective clinics will be provided with a-Bahasa Indonesia plain language statement (PLS) and consent forms. A minimum of two GPs, two midwives, two nurses and ten patients in each clinic will be involved. Their informed consent will also be separately sought.

#### Analysis

The data resulted from pilot phase will be analysed as below:

i. Qualitative data: FGD, interviews, and observation data will be analysed thematically. Thematic analysis will apply a mixed of inductive and deductive approaches [30] similar to the analysis steps at the exploratory and development phases.
ii. Clinics and patients’ quantitative data including demographic data, treatment data, referral or medications administered will be analysed descriptively.

### Language validation and participants’ incentives

All phases in this study are conducted in Bahasa Indonesia except for the development phase which will be conducted bilingually in English and Bahasa Indonesia. The interview and FDG guides, including statements tested in the development phase, will be translated into Bahasa Indonesia and be back-translated into English. A quarter of the interview and FGD transcripts will be translated into English and be back-translated by Indonesian native speakers to Bahasa Indonesia for validation purposes.

Participants in this study will be given a gift voucher as a small token of appreciation for their participation in each interview or survey round or FGD. A research participation certificate will also be offered for participants at the development phase.

## Discussion

This study protocol describes the research design for the development of HDP management pathways in Indonesia. It covers the preliminary phase to explore the Indonesian stakeholders’ views about the current HDP management up to the pilot implementation phase in selected primary care clinics to provide evidence of the pathways’ acceptability and feasibility before their actual implementation in practice settings. The scoping review of current international HDP guidelines combined with the results of exploratory phase will be used to inform the development of the proposed HDP pathways and the pilot study phase.

This protocol will add to the current knowledge of translating HDP guidelines into more practical clinical pathways in developing countries’ primary care. The study will complement the previous investigation of HDP guidelines [35, 36] and pathways [37] by comprehensively looking at HDP management in terms of risks, screening, referral and long-term follow up in primary care and contextualising the recommendations in developing countries settings. The developed and piloted HDP management pathways are expected to provide direct benefits for local patients in Indonesia where the HDP management is challenged by facilities, transport barriers and unstandardised treatment from primary care [11]. From the methodological point of view, this study will add evidence of the combination of MRC and PRISM model as guidance for an implementation study and their application in non-western countries.

### Strengths and limitations

The MRC and PRISM framework have provided structured steps to translate evidence into practice. The qualitative methods in the exploratory phase are appropriate to explore the HDP management in primary care where limited evidence is available in the literature. The statements tested in the development phase are robust because they are derived from the results of our review of HDP management guidelines and our interviews with Indonesian primary care stakeholders. Delphi technique at the development phase is an appropriate method to develop consensus between HDP experts because of its flexibility, its ability to offer anonymity to participants and its benefit to minimise bias from dominant experts compared to the other consensus development methods [38–40, 41]. The application of rigorous scoring criteria for approval for each consensus statement is also expected to increase the pathways’ reliability. Lastly, the mixed methods used at the pilot study phase will allow an in-depth evaluation in practice settings as well as to provide recommendations for further scaling up study.

The limitation of this study, however, is the small sample size at the exploratory and development phase [33]. Nonetheless, this study has anticipated the sample limitation by involving participants from various background. Our development phase also does not involve direct meetings with the participants and may limit their interaction to generate ideas in the consensus [33]. However, this limitation has been anticipated by providing participants with the survey’s summary results, and they are able to revise their responses at the next following rounds. Lastly, this study protocol is designed specifically for Indonesia and may not represent the conditions of other developing countries.

## Abbreviations

HDP: Hypertensive disorders of pregnancy
Jamkesmas: Jaminan Kesehatan Masyarakat (Indonesian public insurance for the poor and nearly poor residents)
JKN: Jaminan Kesehatan Nasional (Indonesian public insurance)
MRC: Medical Research Council
PRISM: Practical, Robust, Implementation and Sustainability Model
WHO: World Health Organization
